# Asymptomatic malaria and hepatitis B do not influence cytokine responses of persons involved in chronic sedentary activities

**DOI:** 10.1186/s12879-020-05692-2

**Published:** 2020-12-14

**Authors:** Nsoh Godwin Anabire, Paul Armah Aryee, Zulka Ziblim, Jonathan Suurbaar, Felix Ansah, Gideon Kofi Helegbe

**Affiliations:** 1 West African Centre for Cell Biology of Infectious Pathogens (WACCBIP), University of Ghana, P. O. Box LG 54, Legon, Accra, Ghana; 2 Department of Biochemistry & Molecular Medicine, School of Medicine, University for Development Studies, P. O. Box TL, 1883 Tamale, Ghana; 3 Department of Nutritional Sciences, School of Allied Health Sciences, University for Development Studies, P. O. Box TL, 1883 Tamale, Ghana

**Keywords:** Sedentary lifestyle, Hypertension, Cytokines, Malaria, Hepatitis B

## Abstract

**Background:**

Chronic Sedentary lifestyles have been linked to increased odds of stress, elevated anxiety and diminished wellbeing, inducing cytokine production and predispose to hypertension and other cardiovascular diseases. In endemic areas, *Plasmodium falciparum* and hepatitis B virus (HBV) infections can trigger pro-inflammatory cytokine responses. However, the impact of these infections on cytokine response profiles in individuals engaged in chronic sedentary activities is unknown. This study was aimed at addressing these concerns using a predominantly sedentary population of traders in the Tamale metropolis of Ghana.

**Method:**

Four hundred respondents were categorized, based on their number of working years (< or ≥ 5 years) and number of working hours per day (< or ≥ 10 h), into sedentary (*≥5 years + ≥ 10 h)* and non-sedentary (*≥ 5 years + < 10 h, < 5 years + ≥ 10 h and <  5 years + < 10 h)* groups. The participants were tested for *P. falciparum* and HBV infections using polymerase chain reaction. Blood pressure and cytokines responses were measured. Associations and comparison analysis between variables were determined, and test statistics with *p < 0.05* were considered statistically significant.

**Results:**

Infection status included: un-infected (93.5%), *P. falciparum* mono-infected (1.0%), HBV mono-infected (3.0%) or *P. falciparum* /HBV co-infected (2.5%). Majority of the participants, 57.0% (*n* = 228) were involved in chronic sedentary life style. That notwithstanding, sedentary lifestyle was independent of the infection groups *(χ*^*2*^ *= 7.08, p = 0.629).* Hypertension was diagnosed in 53.8% of respondents and was independent of infection status *(X* ^*2*^ *= 6.33, p = 0.097).* Pro-inflammatory *(TNF-α, IL-1β, IL-6, IL-8 and IL-12)* and anti-inflammatory *(IL-10, IL-7 and IL-13)* cytokine responses were similar among individuals with different sedentary working time and between hypertensive and non-hypertensive individuals *(p > 0.05 for all comparisons).* Among individuals with different infection status, pro-inflammatory (TNF*-α; p = 0.290, IL-1β; p = 0.442, IL-6; p = 0.686, IFN-γ; p = 0.801, IL-8; p = 0.546, IL-12; p = 0.154)* and anti-inflammatory (*IL-10; p = 0.201, IL-7; p = 0.190, IL-13; p = 0.763)* cytokine responses were similar.

**Conclusion:**

Our data suggest that asymptomatic infections of *P. falciparum* and HBV together with a high prevalence of hypertension did not have any significant impact on cytokine response profiles among predominantly sedentary traders in the Tamale metropolis of Ghana.

## Background

Behavioural or lifestyle activities such as unhealthy diet, physical inactivity, smoking and harmful use of alcohol are recognized as important risk factors of cardiovascular diseases (CVDs) [1, 2]. The effects of these behavioural risk factors may show up in individuals as elevated blood pressure or blood glucose or blood lipids, as well as overweight and obesity [1, 2]. Notwithstanding, the involvement of sedentary lifestyle as a risk factor for hypertension and CVDs cannot be overruled [3, 4]. In particular, prolonged sedentary time can impact upon inflammation [5–7], and is associated with elevated pro-inflammatory and reduced anti-inflammatory cytokine responses [5, 8–10]. Cytokine imbalances, specifically a shift towards pro-inflammatory responses, plays a key role in the pathogenesis of hypertension and CVDs [11].

Sub-Saharan African countries are faced with a huge burden of infectious diseases such as malaria and hepatitis B. Particularly, in malaria endemic settings, it is common to find adults with asymptomatic *Plasmodium falciparum* infections who have developed semi-immunity due to exposure to the parasite [12]. Also, it is common to find adults with chronic hepatitis B virus (HBV) infection who experience no disease symptoms [13]. Though current evidence shows no links between *P. falciparum* or HBV and CVDs [14, 15], both pathogenic diseases majorly trigger inflammatory immune responses [16], and could exacerbate inflammatory responses in adults involved in chronic sedentary lifestyle, which may predispose them to hypertension and CVD.

In many endemic countries including Ghana, traders are observed to be engaged in stress related sedentary activities as they sit for long hours as part of their work [17]. This current study evaluates the impact of carriage of HBV and/or *P. falciparum* on cytokine profiles of this group of people and their risk to developing hypertension. The data generated would be useful in informing the implicit link of these infections to the risk of hypertension and CVDs.

## Methods

### Study design, study site and population

A cross-sectional study was conducted from July to September 2017, on traders in the Tamale metropolis, which is the Northern Regional capital of Ghana. Adult traders (18 years and above) who worked predominantly seated were recruited into the study. The Participants were recruited conveniently after close of work, and the numbers obtained within the sampling period was taken as the sample size.

### Data collection and processing

Socio-demographic data (including; age, sex, marital status, educational status, occupational information) and life style behaviours (intake of alcohol, smoking and self-medication) of each study participant was collected after consent was obtained, using a standardized questionnaire (Supplementary file). Qualified enumerators were given a one-day training, and questionnaire was pre-tested among a set of traders who did not participate in the study. The participants were categorized into young adults (18–35 years), middle aged adults (36–55 years) and the aged (above 55 years) [18].

Blood pressure (BP) of each participant was measured using a semi-automated (digital) sphygmomanometer, MaRS® model MS-702 (Mars Medical Products Co., Ltd., Taiwan). The average of two BP readings, first reading taken at the close of work and second reading taken the next morning prior to start of work, was used in the analysis. BP was categorized as normal (< 120/< 80 mmHg), elevated (120–129/< 80 mmHg) or hypertension stage 1 (130–139/ 80–89 mmHg) and hypertension stage 2 (≥140 /≥90 mmHg) [19]. The proxy for assessing chronic sedentary time among the respondents was based on mainly sitting/seated for long hours (beyond the normal working hours, ≥10 h per day), and being involved in the work for a longer period (≥5 years).

### Laboratory investigations

Venous blood sample (5 mL) was collected from each participant by a qualified phlebotomist into an anticoagulant (EDTA) tube, and used for laboratory investigations.

Hemoglobin (Hb) levels of participants were measured using Mission® Plus Hb meter (ACON Laboratories, Inc., San Diego, USA). Anemia was diagnosed by Hb < 12 g/dl for non-pregnant females and Hb < 13 g/dl for males [20].

Participants were screened for malaria and hepatitis B using CareStart™ HRP-2 rapid diagnostic test kit (Access Bio Inc., New Jersey, USA) and hepatitis B surface antigen (HbsAg) test strips (Intec Products Inc., Xiamen, China) respectively. The presence or absence of plasmodium infection in each participant was confirmed using a nested polymerase chain reaction (PCR) assay [21, 22]. The presence or absence of HBV infection in each participant was also confirmed by a previously described PCR assay [23]. The PCR reagents, reaction volumes and cycling conditions were the same as we have previously reported [24].

### Cytokine analysis

HSTCMAG28SPMX13 Milliplex MAP kit (Merck, Darmstadt, Germany) was used to assay serum levels of nine cytokines; tumor necrosis factor alpha (TNF-α), interleukin (IL)-1β, IL-6, interferon gamma (IFN-γ), IL-12, IL-8, IL-10, IL-7, and IL-13, following the manufacturer’s protocol. The quantification was done on the xMAP Technology platform (Luminex Corporation, Austin, USA). The values of the quality controls and standards were within the ranges provided by the kit. Samples were assayed in duplicates and those with percentage coefficient of variation < 15% were included in the data analysis.

### Statistical analysis

Microsoft Excel 2016 (Microsoft Corporation, Redmond, USA) was used for data entry and proofreading. SPSS Version 20 (IBM Corporation, Chicago, USA) and GraphPad Prism 6 (GraphPad Software Inc.) were used to analyze the data. Median and interquartile range (IQR) was used to present continuous variables and comparisons were done by Krulskal-Wallis or Mann–Whitney test. On the other hand, counts and percentages were used to describe categorical variables, and comparison analysis were done using Fisher’s exact test or Pearson’s chi-square test. *P*-value < 0.05 was considered as statistically significant for all test statistics.

## Results

### Questionnaire response rate and socio-demographic information

The questionnaire was administered to a total of 400 traders. All the participants responded to the questionnaire (100% response rate). Majority (46.0%, *n* = 224) of the respondents were young adults (18–35 years, Additional file 1). Also, 78.5% (*n* = 314) of them were married, and majority (64.5%, *n* = 258) had attained some level of formal education (Additional file 1).

### Behavioral/life style determinants among study participants

Majority (82.5% *n* = 330) of respondents were involved in trading for ≥5 years (Table 2). Many (69.8%, *n* = 279) worked, predominantly sitting/seated beyond the normal work hours per day (≥10 h, Table 2). A smaller proportion of the participants responded to intake of alcohol (3.0%. *n* = 12) and smoking (2.5%. *n* = 10) (Additional file 2). Though all the participants responded to not taking any medications or drugs in the last month prior to the study, more than half (50.5%. *n* = 202) were in the habit of self-medication whenever they were sick such as having chest and bodily pains (Additional file 2). The self-medicated drugs were mainly antimalarials and pain killers. Close to a half (44.5%, *n* = 178) of the respondents were diagnosed with anemia (Supplementary Table 2). Also, more than half were diagnosed with hypertension (type 1 and 2) (53.8%, *n* = 215, Additional file 2).

### Sedentary and infection groups

The participants were categorized into 4 groups based on their number of working years (< or ≥ 5 years) and number of working hours per day (< or ≥ 10 h). The predominantly sedentary participants included those in *≥5 years + ≥ 10 h group* (57.0%, *n* = 228) and the non-sedentary group included those in *≥5 years + < 10 h* group (25.5%, *n* = 102), *< 5 years + ≥ 10 h* group (12.8%, *n* = 51), and *<  5 years + < 10 h* group (4.8%, *n* = 19). Based on the PCRs, the infection status of the participants included un-infected (93.5%, *n* = 374), *P. falciparum* mono-infected (1.0%, *n* = 4), HBV mono-infected (3.0%, *n* = 12) or *P. falciparum*/HBV co-infected (2.5%, n = 10). In the sedentary group, 212 (53.0%) were un-infected, 2 (0.5%) had *P. falciparum* mono-infection, 7 (1.8%) had HBV mono-infection while 7 (1.8%) had *P. falciparum*/HBV co-infection (Table 1*)*. Sedentary lifestyle was independent of the infection groups *(χ*^*2*^ *= 7.08, p = 0.629,* Table 1*).*

**Table 1:** Association between Sedentary life style and Infection status

Infection status	Sedentary and non-sedentary groups				(***X***^***2***^), *p
	> = 5 years + > = 10 h n (%)	> = 5 years + < 10 h n (%)	< 5 years + > = 10 h n (%)	< 5 years + < 10 h n (%)	
Un-infected	212 (53.0)	96 (24.0)	50 (12.5)	16 (4.0)	7.08, *0.629
*P. falciparum mono-infection*	2 (0.5)	1 (0.3)	0 (0.0)	1 (0.3)	
*HBV mono-infection*	7 (1.8)	3 (0.8)	1 (0.3)	1 (0.3)	
*Co-infected*	7 (1.8)	2 (0.5)	0 (0.0)	1 (0.3)	

* analyzed using Pearson’s chi-square test or Fisher’s exact test

χ^2^ = Chi-square value

*p* significant at < 0.05 (2-tailed)

n (%): numbers and proportions

### Socio-demographic information and determinants of stress among infection groups

The proportions of young adults, middle age adults and the aged were similar among the different infection statuses *(χ*^*2*^ *= 12.17, p = 0.058,* Table 2*)*. Similarly, no differences in sex *(χ*^*2*^ *= 4.89, p = 0.108)*, marital status *(χ*^*2*^ *= 6.98, p = 0.073)*, and educational status *(χ*^*2*^ *= 3.51, p = 0.320)* were observed among the different infection statuses (Table 2). The proportions of participants with life style factors such as intake of alcohol *(χ*^*2*^ *= 0.86, p = 0.835)*, smoking *(χ*^*2*^ *= 3.49, p = 0.321)* and resort to self-medications *(χ*^*2*^ *= 1.70, p = 0.636)* were also similar among individuals with different infection status (Table 2). Anemia *(χ*^*2*^ *= 4.03, p = 0.258)* and hypertension *(χ*^*2*^ *= 6.33, p = 0.097)* were also independent of infection status (Table 2).

**Table 2:** Differences in socio-demographic variables and stress determinants among infection groups

Category	Sub-category	Infection status				(***X***^***2***^), *p
		Un-infected n (%)	*P. falciparum mono-infection* n (%)	*HBV mono-infection* n (%)	*Co-infected* n (%)	
**Socio-economic variables**						
**Age**	18–35	202 (54.0)	4 (100.0)	8 (66.7)	10 (100.0)	12.17, *0.058
	36–55	124 (33.2)	0 (0.0)	3 (25.0)	0 (0.0)	
	56^+^	48 (12.8)	0 (0.0)	1 (8.3)	0 (0.0)	
**Sex**	Male	255 (68.2)	3 (75.0)	9 (75.0)	10 (100.0)	4.89, *0.180
	Female	119 (31.8)	1 (25.0)	3 (25.0)	0 (0.0)	
**Marital Status**	Yes	296 (79.1)	1 (25.0)	9 (75.0)	8 (80.0)	6.98, *0.073
	No	78 (20.9)	3 (75.0)	3 (25.0)	2 (20.0)	
**Formal education**	Yes	239 (63.9)	4 (100.0)	7 (58.3)	8 (80.0)	3.51, *0.320
	No	135 (36.1)	0 (0.0)	5 (41.7)	2 (20.0)	
**Life style determinants**						
**Alcohol intake**	Yes	12 (3.0)	0 (0.0)	0 (0.0)	0 (0.0)	0.86, *0.835
	No	388 (97.0)	4 (100.0)	12 (100.0)	10 (100.0)	
**Smoking**	Yes	21 (5.6)	1 (25.0)	1 (8.3)	0 (0.0)	3.51, *0.320
	No	353 (94.4)	3 (75.0)	11 (91.7)	10 (100.0)	
**Self-medication**	Yes	186 (49.7)	2 (50.0)	8 (66.7)	6 (60.0)	1.70, *0.636
	No	188 (50.3)	2 (50.0)	4 (33.3)	4 (40.0)	
**Diagnosed anemia and hypertension**						
**Anemia**	Yes	170 (45.5)	0 (0.0)	4 (33.3)	4 (40.0)	4.03, *0.258
	No	204 (54.5)	4 (100.0)	8 (66.7)	6 (60.0)	
**Hypertensive**	Yes	254 (67.9)	4 (100.0)	5 (41.7)	8 (80.0)	6.33, *0.097
	No	120 (32.1)	0 (0.0)	7 (58.3)	2 (20.0)	

^*^ analyzed using Pearson’s chi-square test or Fisher’s exact test

χ^2^ = Chi-square value

*p* significant at < 0.05 (2-tailed)

n (%): numbers and proportions

### Cytokine responses between sedentary and non-sedentary groups

*To evaluate the impact of chronic sedentary lifestyle on serum cytokine response levels, pro- and anti-inflammatory cytokine responses were compared between the sedentary group;* ≥5 years + ≥ 10 h *group (n = 24) and the non-sedentary groups;* ≥ 5 years + < 10 h *group (n = 6),* < 5 years + ≥ 10 h *group (n = 4), and* <  5 years + < 10 h *group (n = 4). The comparison analysis showed similar levels of pro-inflammatory (*TNF-α; *p* = 0.405, IL-1β; *p* = 0.759, IL-6; *p* = 0.608, IFN-γ; *p* = 0.890, IL-8; *p* = 0.773, IL-12; *p* = 0.667; Table 2*) and anti-inflammatory (*IL-10; *p* = 0.342, IL-7; *p* = 0.222, IL-13; *p* = 0.927; Table 3*) cytokine responses across the four groups.*

**Table 3:** Comparison of cytokine responses across different lifestyle groups

Parameters (pg/mL)	Groups of traders based on number of working years and working hours per day				*p
	≥5 years + ≥ 10 h group	≥ 5 years + < 10 h group	< 5 years + ≥ 10 h group	< 5 years + < 10 h group	
Pro-inflammatory cytokines			Median (IQR), n		
TNF-α	12.12 (9.54–15.38) 20	11.35 (9.65–11.92) 5	12.69 (6.91–14.51) 4	7.13 (4.50–12.99) 4	0.405
IL-1β	3.47 (2.82–4.82) 15	3.52 (2.59–5.503) 4	4.56 (3.30–5.73) 3	3.37 (1.97–4.56) 3	0.759
IL-6	34.88 (25.83–47.90) 20	28.41 (23.06–36.30) 5	39.55 (21.62–52.94) 4	25.32 (21.62–131.3) 4	0.608
IFN-γ	6.355 (3.94–9.83) 18	5.39 (3.73–8.44) 5	7.34 (3.69–10.77) 3	7.95 (3.01–14.20) 4	0.890
IL-8	14.21 (10.32–36.89) 13	12.16 (4.09–20.22) 2	35.94 (11.06–404.4) 4	15.93 (3.01–24.02) 3	0.773
IL-12	4.328 (3.07–5.73) 18	4.27 (3.45–6. 87) 5	2.161 (1.79–5.86) 3	3.872 (1.82–5.96) 3	0.667
Anti-inflammatory cytokines			Median (IQR), n		
IL-10	28.3 (14.17–52.01) 18	16.65 (14.91–50.76) 4	13.41 (5.69–29.60) 4	17.6 (15.06–27.41) 3	0.342
IL-7	17.72 (14.41–23.66) 21	11.83 (10.38–24.46) 5	17.98 (11.91–35.01) 4	10.93 (6.76–17.98) 4	0.222
IL-13	27.17 (19.65–35.52) 15	27.47 (15.37–42.98) 5	23.26 (13.22–34.90) 4	26.27 (15.59–39.87) 4	0.927

*; analyzed by Krulskal-Wallis test

*p* significant at < 0.05 (2-tailed)

*IQR* interquartile range

*n* number of samples

### Cytokine profiles among participants diagnosed with hypertension or anemia

The influence of hypertension or anemia on serum cytokine profiles among the participants was evaluated. Comparison analysis showed similar levels of pro-inflammatory (TNF-α, IL-1β, IL-6, IFN-γ, IL-8 and IL-12) and anti-inflammatory (IL-10, IL-7 and IL-13) cytokine responses between hypertensive and non-hypertensive participants and between anemic and non-anemic participants *(p > 0.05 for all comparisons,* Table 4*)*.

**Table 4:** Comparison of cytokine responses among participants with hypertension or anemia

Cytokines (pg/mL)	Category	Hypertension			Anemia		
		n	Median (IQR)	^*#*^*p*	N	Median (IQR)	^*#*^*p*
TNF-α	Yes	22	11.48 (9.75–14.91)	0.281	13	11.44 (10.28–14.77)	0.573
	No	11	9.91 (6.74–12.95)		20	11.62 (6.82–13.69)	
IL-1β	Yes	19	3.55 (2.82–5.28)	0.563	11	3.91 (3.30–5.28)	0.123
	No	6	3.39 (2.66–4.57)		14	3.03 (2.377–4.57)	
IL-6	Yes	21	40.64 (27.78–47.54)	0.113	13	37.09 (27.78–43.53)	0.957
	No	12	27.26 (21.49–37.50		20	30.43 (23.26–52.17)	
IFN-γ	Yes	21	6.62 (3.96–10.12)	0.378	12	7.12 (5.03–9.38)	0.409
	No	9	6.89 (3.59–7.41)		18	6.26 (3.60–8.96)	
IL-8	Yes	16	15.44 (10.25–56.46)	0.449	8	15.44 (10.95–51.21)	0.764
	No	6	12.79 (8.72–21.6)		14	13.7 (9.01–29.86)	
IL-12	Yes	21	4.01 (2.81–5.82)	0.989	13	4.47 (3.35–5.94)	0.519
	No	8	4.54 (2.95–5.60)		16	3.99 (2.72–5.35)	
IL-10	Yes	20	24.62 (16.57–47.64)	0.220	12	20.55 (5.21–40.69)	0.922
	No	9	14.39 (10.33–61.24)		17	24.37 (13.52–51.11)	
IL-7	Yes	22	18.16 (11.7–22.06)	0.582	13	19.24 (17.11–23.66)	0.082
	No	12	14.41 (11.45–23.63)		21	13.99 (10.38–21.45)	
IL-13	Yes	19	27.47 (20.06–37.07)	0.159	13	29.9 (21.79–38.55)	0.205
	No	9	17.68 (13.24–34.71)		15	25.36 (14.89–34.08)	

^***#***^; analyzed by Mann-Whitney U test at < 0.05 level of statistical significance

*p* significant at < 0.05 (2-tailed)

*IQR* interquartile range

*n* number of samples

### Cytokine profiles among infection groups

To gain insight into the possible influence of asymptomatic carriage of *P. falciparum* and/or HBV on cytokine responses among the participants, the cytokine responses were compared across the infection statuses. Serum levels of pro-inflammatory cytokines were similar among the participants with different infection status (*TNF-α; p = 0.290, IL-1β; p = 0.442, IL-6; p = 0.686, IFN-γ; p = 0.801, IL-8; p = 0.546, IL-12; p = 0.154;* Table 4). Similarly, no differences in anti-inflammatory cytokine responses were observed across the infection statuses (*IL-10; p = 0.201, IL-7; p = 0.190, IL-13; p = 0.763;* Table 5). Next, the participants stratified into four groups as: those involved in sedentary lifestyle and infected with *P. falciparum* and/or HBV infections, those involved in sedentary life style and Un-infected, those involved in non-sedentary lifestyle and infected with *P. falciparum* and/or HBV infections, and those involved in non-sedentary life style and Un-infected. Comparison analysis showed that levels of the pro- and anti-inflammatory cytokine levels were similar across the groups *(p > 0.05 for all comparisons,* Table 6*)*.

**Table 5:** Comparison of serum cytokines levels across the different infection groups

Parameters (pg/mL)	Infection status				*P
	Un-infected	*P. falciparum mono-infection*	*HBV mono-infection*	*P. falciparum/HBV co-infection*	
Pro-inflammatory cytokines			Median (IQR), n		
TNF-α	11.52 (8.55–13.85) 11	8.26 (4.49–26.63) 4	11.44 (6.93–12.56) 9	12.95 (10.97–15.59) 9	0.290
IL-1β	4.56 (2.67–6.34) 9	2.94 (1.97–4.14) 3	3.07 (2.65–4.09) 6	3.55 (3.12–4.82) 7	0.442
IL-6	37.09 (24.44–48.26) 11	32.13 (19.09–165.8) 3	28.70 (22.68–39.35) 9	34.52 (37.59–55.42) 10	0.686
IFN-γ	6.00 (3.18–8.30) 10	7.79 (3.22–16.00) 3	6.99 (5.70–9.38) 8	6.10 (3.74–9.85) 9	0.801
IL-8	47.38 (7.71–395.00) 9	10.17 (5.51–20.22) 3	12.99 (10.54–19.48) 5	15.93 (9.15–23.60) 5	0.546
IL-12	2.97 (1.82–5.02) 10	3.87 (2.87–4.00) 3	4.54 (3.75–5.12) 7	5.68 (3.93–6.32) 9	0.154
Anti-inflammatory cytokines			Median (IQR), n		
IL-10	17.18 (11.82–25.67) 9	24.86 (15.06–49.26) 3	23.20 (11.86–47.39) 8	27.41 (17–99.52) 9	0.201
IL-7	16.67 (11.33–21.49) 11	10.40 (6.70–18.81) 4	17.54 (11.85–23.66) 9	19.50 (16.79–25.00) 10	0.190
IL-13	19.65 (15.86–35.49) 9	27.47 (25.36–41.54) 3	25.55 (13.11–38.54) 8	31.50 (21.60–35.35) 8	0.763

*; analyzed by Krulskal-Wallis test at < 0.05 level of statistical significance

*p* significant at < 0.05 (2-tailed)

*IQR* interquartile range

*n* number of samples

**Table 6:** Comparison of serum cytokines levels across sedentary and non-sedentary groups with different infections

Parameters (pg/mL)	Sedentary groups with different infection status				*p
	Sedentary and Infected group	Sedentary and Un-infected	Non-sedentary and Infected group	Non-sedentary and Un-infected group	
Pro-inflammatory cytokines			Median (IQR), n		
TNF-α	12.95 (10.03–15.59) 13	10.72 (8.55–12.89) 7	9.84 (6.45–11.92) 9	12.69 (7.93–14.51) 4	0.349
IL-1β	3.35 (2.91–4.67) 10	3.91 (1.98–9.58) 5	3.14 (2.36–4.59) 6	4.56 (3.62–5.44) 4	0.615
IL-6	37.63 (26.48–50.68) 13	33.21 (25.49–47.9) 8	27.78 (20.98–36.3) 9	42.01 (21.21–56.59) 3	0.534
IFN-γ	6.89 (3.83–10.18) 17	6.00 (3.86–7.47) 8	6.25 (3.87–8.81) 12	5.52 (2.16–9.92) 4	0.867
IL-8	13.6 (10.25–19.34) 8	47.38 (7.60–395.00) 5	15.93 (7.36–22.12) 5	35.95 (5.36–404.4) 4	0.685
IL-12	4.68 (3.78–5.83) 12	4.12 (2.75–5. 86) 7	4.27 (3.87–5.96) 7	1.99 (1.80–3.54) 4	0.119
Anti-inflammatory cytokines			Median (IQR), n		
IL-10	34.4 (23.94–69.84) 13	12.66 (11.82–35.28) 5	16.49 (14.39–27.41) 7	17.37 (11.53–29.71) 4	0.098
IL-7	20.22 (17.72–26.28) 7	17.04 (11.92–20.51) 8	19.24 (10.46–19.76) 3	17.81 (10.53–34.92) 4	0.461
IL-13	30.14 (25.00–36.64) 10	19.65 (15.86–48.81) 5	27.47 (14.63–38.20) 9	23.26 (15.70–34.90) 4	0.852

*; analyzed by Krulskal-Wallis test at < 0.05 level of statistical significance

*p* significant at < 0.05 (2-tailed)

*IQR* interquartile range

*n* number of samples

## Discussion

This current study evaluates the influence of carriage of HBV and *P. falciparum* infections on cytokine profiles of predominantly sedentary traders in the Tamale metropolis of Ghana. This is from a viewpoint that sedentary activities of traders can predispose them to CVDs [3, 4] . Amidst various reasons, predisposition to hypertension and CVDs could be ascribed to induction of inflammatory mediators, and strong evidence suggests that prolonged sedentary time impacts upon inflammation [5–7]. Since HBV and *P. falciparum* infections majorly trigger pro-inflammatory cytokine responses, they could be viewed to increase the risk of hypertension in persons involved in chronic sedentary behaviour.

The study established chronic sedentary behaviour as common among the traders, nevertheless, the behaviour had no significant impact on the cytokine profiles of the traders. In particular, prolonged sedentary time is associated with increased pro-inflammatory cytokine responses including, TNF-α, IL-6 and IL-8 [5, 8, 10], and these cytokines are observed to play key roles in the pathogenesis of some CVDs [11]. Also, prolonged sedentary time could be associated with reduced anti-inflammatory cytokine responses including IL-10 [9]. This assertion is buttressed by the association of sedentary lifestyle with reduced peripheral circulation of a key anti-inflammatory molecule, adiponectin [5], and particularly, adiponectin induces the release of IL-10 by human leukocytes [25]. However, evidence shows that disruption of prolonged sitting with short bouts of physical activity has beneficial effects including reducing inflammatory mediators in individuals [8, 26]. The participants in this study, though are a predominantly sedentary group, their sitting time may be intermittently interrupted as they get up to sell items and perform some mobile trading activities. Therefore, it is possible that such mobility may account for the similarity in cytokine responses among the traders categorized into the different sedentary lifestyle.

The study reports a 53.8% prevalence of hypertension among the traders, which is higher than the range of 19.0 to 48.0% reported nationally [27]. The evidence of hypertension among the traders corroborates the finding of a previous study conducted among market men and women in the Tamale metropolis of Ghana [3]. What is worrying from this study is the evidence that majority of the participants’ resort to self-medication when they felt bodily and chest pains; which are known symptoms of hypertension. There is therefore the need for concerted efforts to raise knowledge and awareness of hypertension among predominantly sedentary traders in the Tamale Metropolis of Ghana. Though hypertension was common among the participants, it was independent of *P. falciparum* and/or HBV infections, which may lend support to the evidence that have shown no association between *P. falciparum* or HBV with CVDs [14, 15]. Unlike the study that showed an imbalance in serum pro- and anti-inflammatory cytokine responses in individuals with hypertension [28], this current study observed a contrary finding. We speculate that differences in the etiology of hypertension and population-specific factors may contribute to our current finding.

The prevalence of anemia (44.5%) reported among the traders is slightly higher than the national rate of 42.0% previously reported for non-pregnant/non-breastfeeding individuals [29]. Evidence shows that poorer or lack of exercise is a risk factor for anemia [30], which may explain our finding of a substantial number of the traders with anemia, since most of the traders spent longer hours sitting. This anemia may lead to prolonged sedentary time among the traders as they are likely to feel lethargic upon exertion. It has also been reported that anemia is a marker of an increased risk for hypertension and CVD-related deaths [31, 32]. Much as this call for caution among sedentary traders, there is the need for routine diagnosis of anemia among risks groups including traders, so as to avert the possible risks of developing CVDs and subsequent death.

Though *P. falciparum* and HBV majorly trigger the release of the pro-inflammatory cytokines [16], the infections appeared not to markedly influence the inflammatory responses in the traders. It has been suggested that adults with *P. falciparum* infection show controlled inflammatory responses due to increased history of exposure, and that higher parasite loads are required to elicit the inflammatory responses and cause disease symptoms [33]. HBV infection on the other hand has different phases, and during the immune tolerance phase, the host elicits no vigorous inflammatory response to the infection and so no disease symptoms are observed [13]. It is very likely that the traders who were domiciled in an endemic region for both conditions were historically increasingly exposed and were also more likely to be in the immune tolerance phase for the two conditions respectively, and this could account for the independence of infections on the cytokine responses.

### Limitation

A smaller sample size was used and our observation should be replicated in a substantially larger population. The measurement of sedentary behaviour was based on self-report, per the questionnaire administered. Consequently, the use of accelerometers could have offered an increased accuracy in the measurement of sedentary time. Nonetheless, time spent in self-reported sedentary behaviour has been recognized as a unique risk factor for several cardio-metabolic diseases [10, 34]. Among the study participants, the stage of HBV or *P. falciparum* infection was not known. The study described the infections as asymptomatic based on the results of the RDTs and PCRs, and coupled with the observations that the study participants went about their routine activities with no experience of disease symptoms.

## Conclusion

Our data suggest that asymptomatic infections of *P. falciparum* and HBV as well as a high prevalence of hypertension and anemia had no significant impact on cytokine response profiles among predominantly sedentary traders in Tamale metropolis of Ghana. In addition, the infections did not influence the occurrence of hypertension.

## Supplementary Information

**Additional file 1. **Socio-demographic characteristics of the study participants (*n* = 400).

**Additional file 2. **Behavioral/life style determinants in study participants (*n* = 400).

## Data Availability

The datasets supporting the findings of this article are available in this manuscript.

## Abbreviations

BP: Blood pressure
CVDs: Cardiovascular diseases
Hb: Hemoglobin
HbsAg: Hepatitis B surface antigen
HBV: Hepatitis B virus
IFN-γ: Interferon gamma
IL: Interleukin
IQR: Interquartile range
PCR: Polymerase chain reaction
RDTs: Rapid diagnostic tests
TNF-α: Tumor necrosis factor alpha
