# Supplementary material for: Asymptomatic malaria and hepatitis B do not influence cytokine responses of persons involved in chronic sedentary activities
Source: BMC Infect Dis. 2020 Dec 14;20:957. doi: 10.1186/s12879-020-05692-2 (PMC7737354; doi:10.1186/s12879-020-05692-2)
Supplement: Supplementary file 1 — Additional file 1. Socio-demographic characteristics of the study participants (n = 400). [file 12879_2020_5692_MOESM1_ESM.docx]

**Additional file 1. Socio-demographic characteristics of the study participants (n=400)**

| Category | | Sub-category | n (%) | 95% CI |
| --- | --- | --- | --- | --- |
| Age | | 18-35 | 224 (56.0) | 204, 243 |
|  |  | 36-55 | 127 (31.8) | 109, 146 |
|  |  | 56^+^ | 49 (12.2) | 37, 63 |
| Sex | | Male | 277 (69.3) | 258, 294 |
|  |  | Female | 123 (30.8) | 106, 142 |
| Marital Status |  | Married | 314 (78.5) | 297, 329 |
|  |  | Single | 76 (19.0) | 62, 92 |
|  |  | Divorced | 10 (2.5) | 5, 18 |
| Formal education | | None | 142 (35.5) | 124, 161 |
|  |  | Basic | 121 (30.3) | 104, 140 |
|  |  | Secondary | 92 (23.0) | 76, 109 |
|  |  | Tertiary | 45 (11.2) | 34, 58 |

n (%): counts and proportions

(95% CI): 95% confidence intervals of counts
