# Supplementary material for: Asymptomatic malaria and hepatitis B do not influence cytokine responses of persons involved in chronic sedentary activities
Source: BMC Infect Dis. 2020 Dec 14;20:957. doi: 10.1186/s12879-020-05692-2 (PMC7737354; doi:10.1186/s12879-020-05692-2)
Supplement: Supplementary file 2 — Additional file 2. Behavioral/life style determinants in study participants (n = 400). [file 12879_2020_5692_MOESM2_ESM.docx]

**Additional file 2. Behavioral/life style determinants in study participants (n=400)**

| Category | | Sub-category | n (%) | 95% CI |
| --- | --- | --- | --- | --- |
| Years in occupation | | <5 | 70 (17.5) | 56, 86 |
|  |  | ≥5 | 330 (82.5) | 314, 344 |
| Hours at work per day | | <10 | 121 (30.2) | 104, 140 |
|  |  | ≥10 | 279 (69.8) | 260, 296 |
| Alcohol intake | | Yes | 12 (3.0) | 7, 20 |
|  |  | No | 388 (97.0) | 380, 393 |
| Smoking |  | Yes | 23 (5.8) | 15, 33 |
|  |  | No | 377 (94.3) | 367, 385 |
| Self-medication | | Yes | 202 (50.5) | 182, 222 |
|  |  | No | 198 (49.5) | 178, 218 |
| Anemia | | Yes | 178 (44.5) | 159, 198 |
|  |  | No | 222 (55.5) | 202, 241 |
| Blood pressure | | Normal | 129 (32.3) | 111, 148 |
|  |  | Elevated | 56 (14.0) | 43, 71 |
|  |  | hypertension stage 1 | 123 (30.8) | 106, 142 |
|  |  | hypertension stage 2 | 92 (23.0) | 76, 109 |

n (%): counts and proportions

(95% CI): 95% confidence intervals of counts
